# Chiral Mn^III^ (Salen) Immobilized on Organic Polymer/Inorganic Zirconium Hydrogen Phosphate Functionalized with 3-Aminopropyltrimethoxysilane as an Efficient and Recyclable Catalyst for Enantioselective Epoxidation of Styrene

**DOI:** 10.3390/polym11020212

**Published:** 2019-01-26

**Authors:** Xiaochuan Zou, Yue Wang, Cun Wang, Kaiyun Shi, Yanrong Ren, Xin Zhao

**Affiliations:** 1 Department of Biological and Chemical Engineering, Chongqing University of Education, Chongqing 400067, China; zxcvip2003@163.com (X.Z.); b1083@163.com (Y.W.); wangcun5224@126.com (C.W.); shiky@cque.edu.cn (K.S.); renyr@cque.edu.cn (Y.R.); 2 Chongqing Collaborative Innovation Center of Functional Food, Chongqing University of Education, Chongqing 400067, China; 3 Chongqing Engineering Technology Research Center for Functional Food, Chongqing University of Education, Chongqing 400067, China; 4 Chongqing Engineering Laboratory of Functional Food, Chongqing University of Education, Chongqing 400067, China

**Keywords:** chiral Mn^III^ (salen) complex, 3-aminopropyltrimethoxysilane-functionalized, organic polymer/inorganic zirconium hydrogen phosphate, asymmetric epoxidation

## Abstract

Organic polymers/inorganic zirconium hydrogen phosphate (ZSPP, ZPS-IPPA, and ZPS-PVPA) functionalized with 3-aminopropyltrimethoxysilane were prepared and used to support chiral Mn^III^ (salen) complexes (Jacobsen’s catalyst). Different characterization methods demonstrated that the chiral Mn^III^ (salen) complexes was successfully supported on the surface of the carrier (ZSPP, ZPS-IPPA, or ZPS-PVPA) through a 3-aminopropyltrimethoxysilane group spacer. The supported catalysts effectively catalyzed epoxidation of styrene with *m*-chloroperbenzoic acid (*m*-CPBA) as an oxidant in the presence of *N*-methylmorpholine *N*-oxide (NMO) as an axial base. These results (*ee*%, 53.3–63.9) were significantly better than those achieved under a homogeneous counterpart (*ee*%, 46.2). Moreover, it is obvious that there was no significant decrease in catalytic activity after the catalyst 3 was recycled four times (*cons*%: from 95.0 to 92.6; *ee*%: from 64.7 to 60.1). Further recycles of catalyst 3 resulted in poor conversions, although the enantioselectivity obtained was still higher than that of corresponding homogeneous catalyst even after eight times. After the end of the eighth reaction, the solid catalyst was allowed to stand in 2 mol/L of dilute hydrochloric acid overnight, prompting an unexpected discovery that the catalytic activity of the catalyst was recovered again at the 9th and 10th cycles of the catalyst.

## 1. Introduction

Styrene epoxide is a very important class of intermediate that is widely used in the fields of medicine, pesticides, and other optical materials [1,2]. The chiral homogeneous Mn^III^ (salen) complex [3,4] has been proven to be one of the most excellent catalysts for enantioselective epoxidation of styrene. However, after the end of the reaction, there are still difficulties in separating the catalyst from the product; the catalyst is difficult to recover and recycle. In order to address this issue, many research groups have attempted to immobilize chiral homogeneous Mn^III^ (salen) complexes onto/into various solid supports [5,6,7,8,9,10,11,12,13,14] by covalent or axial methods. Some representative supports include MCM-41, LDHS, silica, polymers, dendrimer, graphene oxide, glass beads, etc. Regrettably, despite their excellent performance in separation and reuse, immobilization often decreased enantioselectivity or efficiency of heterogeneous Mn^III^ (salen) complex compared to its homogeneous counterparts. Therefore, novel and effective heterogeneous chiral Mn^III^ (salen) complexes for asymmetric epoxidation of styrene are highly desirable [13].

Recently, a new class of organic/inorganic hybrid materials has received extensive attention due to their simple synthesis and their possession of both organic and inorganic constituents. More importantly, these support materials possess better thermal and mechanical stability during reaction. In previous works, a series of organic polystyrene–inorganic zirconium phosphate hybrid materials, including zirconium oligostyrenylphosphonate-phosphate (ZSPP) [15], zirconium poly(styrene-isopropenyl-phosphonate)-phosphate (ZPS-IPPA) [16], and zirconium poly(styrene-phenylvinyl-phosphonate)-phosphate (ZPS-PVPA) [17,18,19], were reported by our group. These hybrid materials, which were frequently functionalized on the benzene ring in the organic component, have been demonstrated to be a versatile support to immobilize homogeneous chiral Mn^III^ (salen) complexes. The corresponding works demonstrated that ZPS-PVPA-based catalyst can effectively catalyze *α*-methylstyrene and indene, especially *α*-methylstyrene, with a conversion and enantioselectivity of 99%. However, when the substrate is styrene, *ees* were not satisfactory.

Furthermore, no attempts were made using new amino-modified organic polystyrene–inorganic zirconium phosphate hybrid material as catalyst supports, where amino-functionalization occurs on the inorganic zirconium phosphate rather than the organic benzene ring, and none were made using new amino-modified organic polystyrene-inorganic zirconium phosphate hybrid materials for the immobilization of chiral Mn^III^ (salen) complexes. Compared with earlier reports, one of the biggest benefits of the new method is the avoidance of carcinogenic chloromethyl ether during the functionalization of hybrid materials. In addition, the new hybrid material was obtained under mild conditions and had fewer reaction steps during functionalization.

**Scheme 1:**
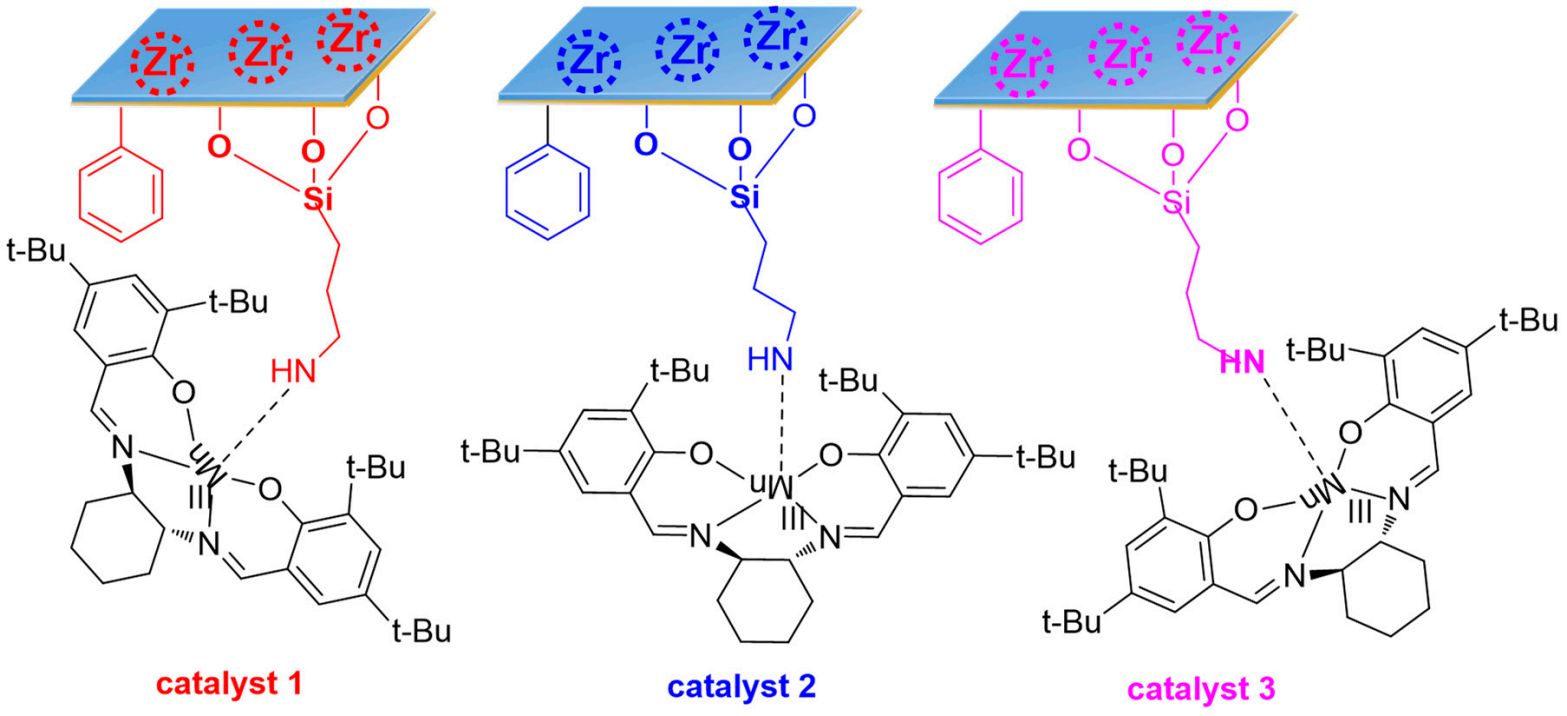
Illustration of catalyst 1, catalyst 2 and catalyst 3.

In order to better understand these new functionalized hybrid materials, and for the sake of searching for more stable, efficient, simple, and reusable heterogenous Mn^III^ (salen) catalysts, we continued to make further efforts. Herein, we extend our attempts to heterogenize chiral Mn^III^ (salen) complexes which was axially immobilized on amino-modified organic polystyrene–inorganic zirconium phosphate hybrid material, in which amino-functionalization occurred on the inorganic zirconium phosphate different from earlier reports (Scheme 1). The results showed that the heterogeneous catalysts efficiently promoted the enantioselective reaction of styrene, producing higher chiral induction (*ee*%, 53.3–63.9) than seen in homogeneous Mn^III^ (salen) complexes (*ee*%, 46.2). In order to study the influence of reaction conditions on the catalytic reaction in more detail, factors such as catalyst dosage, reaction temperature, and solvent were also screened. Moreover, it is obvious that there was no significant decrease in catalytic activity after catalyst 3 was recycled four times (*cons*%: from 95.0 to 92.6; *ee*%: from 64.7 to 60.1). Further recycles of heterogeneous catalyst resulted in poor conversions, although the enantioselectivity obtained was still higher than that of corresponding homogeneous catalyst even after eight times.

## 2. Material and Methods

### 2.1. Materials

(1R,2R)-(−)-1,2-Diaminocyclohexane, *n*-nonane, NMO, and *m*-CPBA were supplied by Alfa Aesar (Tianjian, China). Other commercially available chemicals were laboratory-grade reagents from local suppliers. Styrene was passed through a pad of neutral alumina before use. Chiral salen ligand and chiral Mn^III^ (salen) complex were synthesized according to the literature procedures [3].

### 2.2. Methods

Fourier transform infrared (FT-IR) spectra were recorded from KBr pellets using a Bruker RFS100/S spectrophotometer (Bruker, Karlsruhe, Germany), and diffuse reflectance UV–vis spectra of the solid samples were recorded in the spectrophotometer with an integrating sphere, using BaSO_4_ as standard (Bruker, Karlsruhe, Germany). X-ray photoelectron spectra were recorded on ESCALab250 instrument (Thermo Fisher, Waltham, MA, USA). The Mn contents of the catalysts were determined by TAS-986G atomic absorption spectroscopy (Pgeneral, Beijing, China). Scanning electron microscope (SEM) were performed on SU8010 (Hitachi, Tokyo, Japan) microscopy. Transmission electron microscope (TEM) were obtained on a TECNAI10 (PHILIPS, Amsterdam, The Netherlands) apparatus. Energy Dispersive Spectrometer (EDS) were obtained on a JSM-7800F (JEOL, Beijing, China) apparatus. The interlayer spacings were obtained on a DX-1000 automated X-ray power diffractometer, using Cu Kα radiation and internal silicon powder standard with all samples (Puxi, Beijing, China). The patterns were generally measured between 2.00° and 80.00°, with a step size of 0.02° min^−1^ and X-ray tube settings of 36 kV and 20 mA. The yields (with n-nonane as internal standard) and the *ee* values were analyzed by gas chromatography (GC) with a Shimadzu GC2014 (Shimadzu, Kyoto, Japan) instrument equipped using a chiral column (HP19091G-B233, 30 m × 0.25 mm × 0.25 μm) and (Flame ionization detector) (FID) detector, injector 230 °C, detector 230 °C. The column temperature for styrene was in the range of 80–180 °C. The retention times of the corresponding chiral styrene epoxides are as follows: column temperature of 80 °C, *t*_R_ = 14.7 min, *t*_S_ = 14.9 min.

## 3. Preparation of Catalysts

### 3.1. Synthesis of Organic Polymer/Inorganic Zirconium Hydrogen Phosphate (ZSPP, ZPS-IPPA and ZPS-PVPA)

The synthesis and characterization of ZSPP, ZPS-IPPA, and ZPS-PVPA have been reported earlier by our group [15,16,17].

**Scheme 2:**
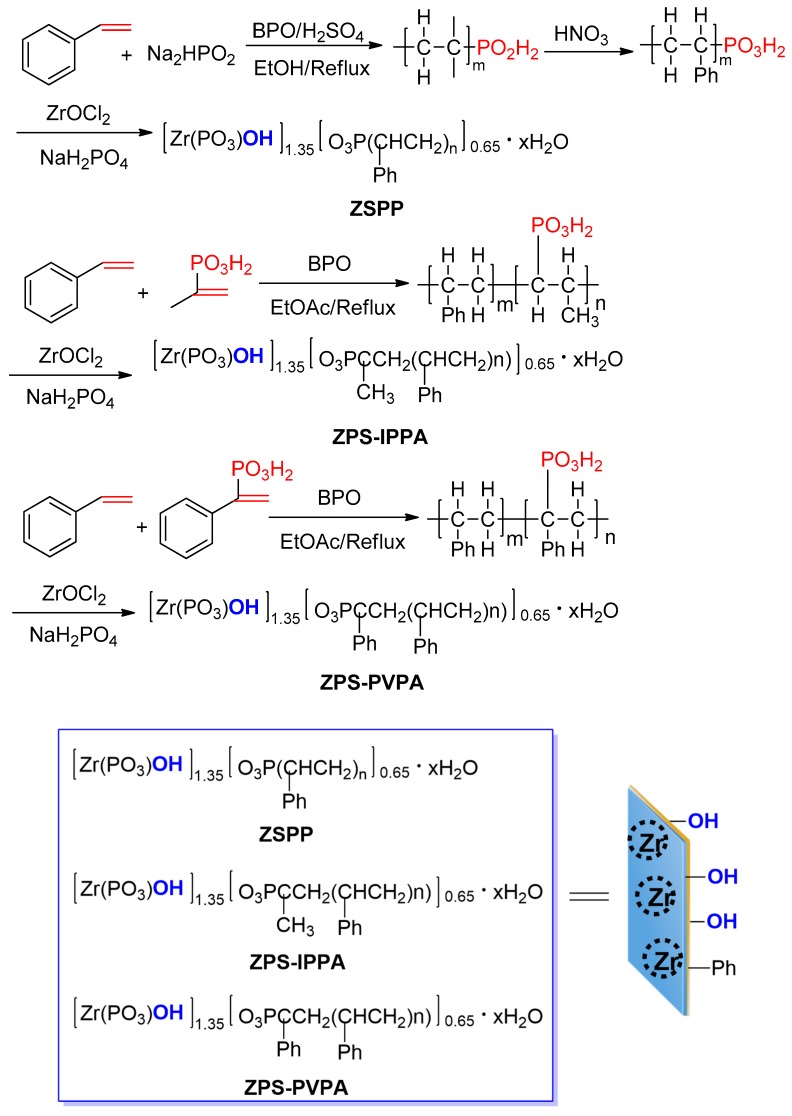
Synthesis of ZSPP, ZPS-IPPA, and ZPS-PVPA.

ZSPP, ZPS-IPPA, and ZPS-PVPA were prepared according to Scheme 2.

### 3.2. Synthesis of Organic Polymer/Inorganic Zirconium Hydrogen Phosphate Functionalized with 3-Aminopropyltrimethoxysilane

**Scheme 3:**
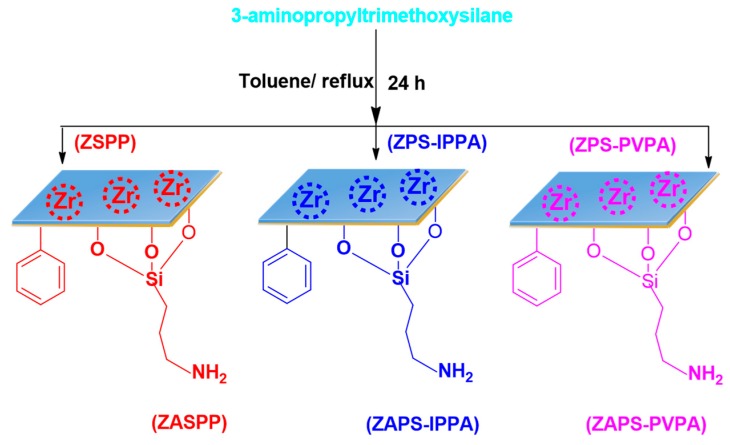
Synthesis of ZASPP, ZAPS-IPPA, and ZAPS-PVPA.

The organic polymer–inorganic zirconium hydrogen phosphate (ZSPP, ZPS-IPPA, or ZPS-PVPA) was added into 50 mL anhydrous toluene, and 1.2 mL of 3-aminopropyltrimethoxysilane was syringed into it. The reaction mixture was then kept at 80 °C for 12 h. After completion of the reaction, it was cooled to room temperature, filtered, washed successively with toluene, water, and ethanol, and dried overnight at 60 °C under vacuum to obtain amine modified organic polymer–inorganic zirconium hydrogen phosphate. The solid products were abbreviated as ZASPP, ZAPS-IPPA, or ZAPS-PVPA (Scheme 3).

### 3.3. Immobilization of Chiral Mn^III^ (Salen) Complex on the ZASPP, ZAPS-IPPA, and ZAPS-PVPA

**Scheme 4:**
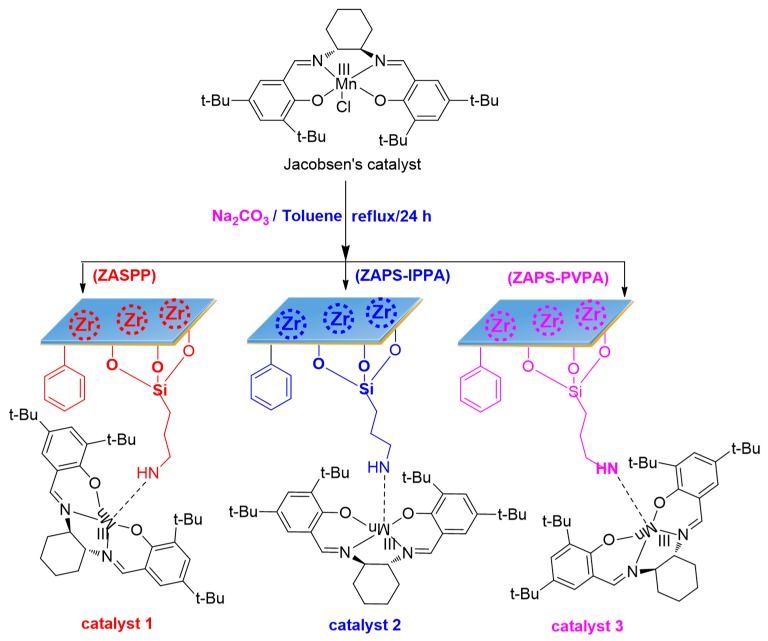
Synthesis of the immobilized catalysts.

In a 50 mL round bottom flask, ZASPP, ZAPS-IPPA, or ZAPS-PVPA (0.2 g) was added, then 10 mL of toluene was immersed in solids, swelled for 30 min, and then chiral Mn^III^ (salen) complex (543 mg, 1.0 mmol), Na_2_CO_3_ (120 mg, 3 mmol) was added to the reaction flask at the same time. After refluxing for 24 h, the reaction was completed. After cooling to room temperature, it was filtered, and then the brown solid was washed successively with ethanol, dichloromethane, and distilled water, and then dried in a vacuum at 60 °C overnight. The CH_2_Cl_2_ filtrate was detected by UV–vis until no peaks could be detected (with CH_2_Cl_2_ as reference). Yield: 96.5%, 96.8%, 97.1%, respectively. The loading of Mn^III^ (salen) complex in the immobilized catalysts, based on the Mn element, was 0.056–0.083 mmol/g, determined by AAS (Scheme 4).

### 3.4. Asymmetric Epoxidation

The catalytic activity of the heterogeneous catalyst was investigated using styrene as a substrate in the *m*-CPBA/NMO system [16]. A solution of styrene (0.5 mmol), NMO (337.5 mg, 2.5 mmol), nonane (internal standard, 90.1 mL, 0.5 mmol), and immobilized Mn^III^ (salen) complexes (0.015 mmol, 3.0 mol%, based on the Mn element) in CH_2_Cl_2_ (3 mL) was cooled to the desired temperature. Solid *m*-CPBA (172.5 mg, 1.0 mmol) was slowly added in four portions over 2 min. After the reaction was completed, the mixture was washed with NaOH (4 mL, 1.0 M) solution and brine to remove *m*-CPBA and dried over anhydrous Na_2_SO_4_. The conversion and *ee* values of styrene epoxide was determined by GC, with *n*-nonane as an internal standard. The racemic sample of the epoxide was independently prepared by the epoxidation of styrene with *m*-CPBA in CHC1_3_ at 0 °C, and detected by GC [18].

## 4. Results and Discussion

### 4.1. FT-IR Spectroscopy

**Figure 1:**
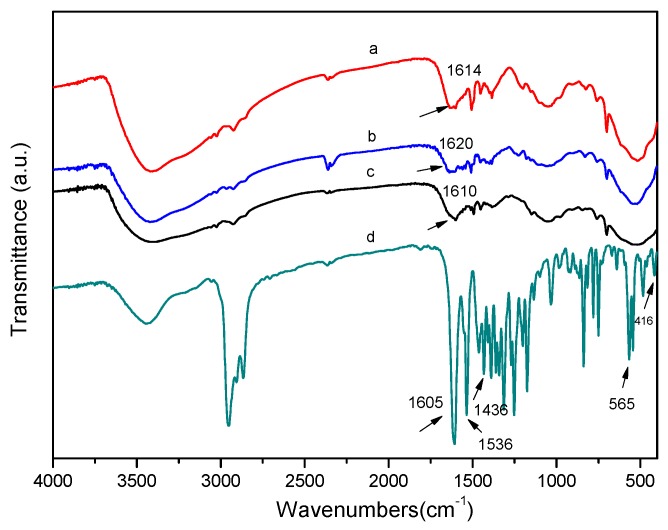
FT-IR spectra of catalysts. (**a**) Catalyst 1, (**b**) catalyst 2, (**c**) catalyst 3, (**d**) chiral Mn^III^ (salen) complex.

The FT-IR spectra of the immobilized Mn^III^ (salen) catalysts and the corresponding homogeneous Mn^III^ (salen) catalyst can be seen in Figure 1. In Figure 1a–c, the spectrum shows intense and large bands in the region of 3200–3600 cm^−1^. These bands are attributed to the surface residue –OH from SiOH and the H_2_O. The bands at near 1026 cm^−1^ mainly belong to Ph–O–C stretching vibrations, which appeared in the supports of ZSPP, ZPS-IPPA, or ZPS-PVPA. In Figure 1d, bands at 1605, 1536, 1436, 565, and 416 cm^−1^ can be identified, which relate to the homogeneous Mn^III^ (salen) catalyst. After grafting the Mn^III^ (salen) onto the 3-aminopropyltrimethoxysilane-functionalized ZSPP, ZPS-IPPA, or ZPS-PVPA, a new characteristic band appeared at around 1615 cm^−1^, which is assigned to the C=N stretching vibration in the Mn^III^ (salen) framework. Obviously, the structure of the Mn^III^ (salen) complex is maintained in the immobilized state [12]. Notably, the characteristic vibrations of C=N slightly shifted from 1605 to 1610–1620 cm^−1^ after grafting, compared with those of the neat complex (Figure 1a–c vs. Figure 1d). The results showed that the chiral Mn^III^ (salen) complex was axially immobilized on the organic polymer–inorganic zirconium hydrogen phosphate through the 3-aminopropyltrimethoxysilane linker.

### 4.2. DR UV–Vis Spectroscopy

**Figure 2:**
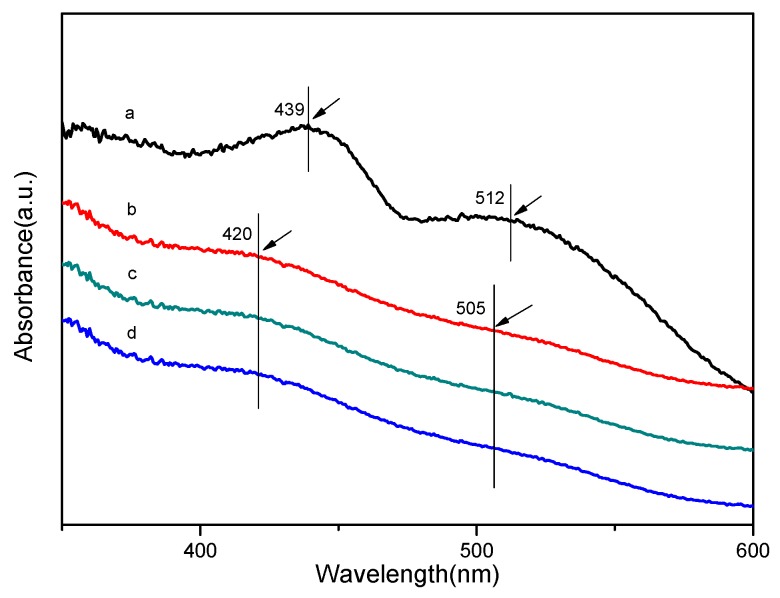
UV–vis spectra catalysts. (**a**) Chiral Mn^III^ (salen) complex, (**b**) catalyst 1, (**c**) catalyst 2, (**d**) catalyst 3.

Figure 2 shows the UV–vis spectra of chiral Mn^III^ (salen) complex and the organic polymer–inorganic zirconium hydrogen phosphate-based catalysts. The UV–vis spectra provides further evidence for the successful grafting, since the characteristic bands of the chiral Mn^III^ (salen) complex at around 439 and 512 nm are slightly shifted to around 420 and 505 nm for the organic polymer–inorganic zirconium hydrogen phosphate-based catalysts, respectively (Figure 2a vs. Figure 2b–d). The band at around 420 nm is attributed to the ligand-to-metal charge-transfer transitions in the chiral Mn^III^ (salen) complex, and the band at around 505 nm belongs to the metal-to-metal charge-transfer band between the chiral Mn^III^ (salen) complexes [17]. The blue shift of the characteristic bands demonstrated that an interaction exists between the heterogeneous catalysts and the 3-aminopropyltrimethoxysilane-modifed organic polymer–inorganic zirconium hydrogen phosphate-based catalyst supports [9].

### 4.3. XRD Analysis

**Figure 3:**
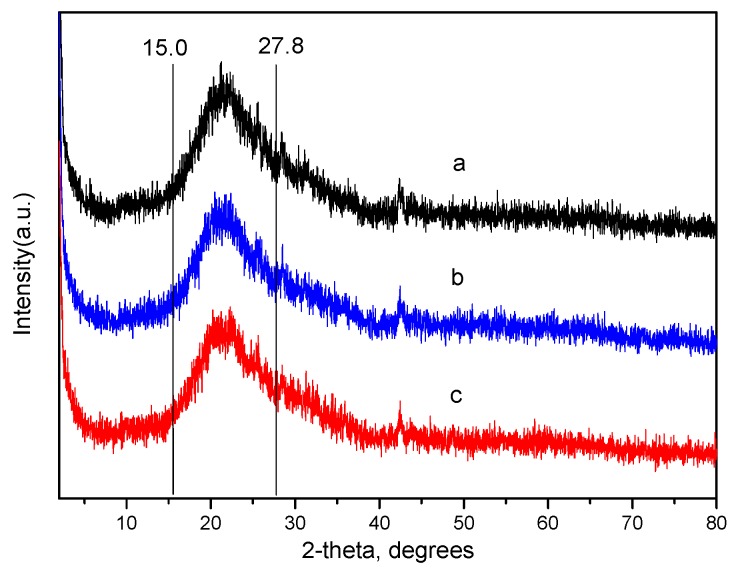
XRD patterns of catalysts (**a**) catalyst 1, (**b**) catalyst 2, (**c**) catalyst 3.

Figure 3 shows X-ray diffraction (XRD) patterns of heterogeneous catalysts 1–3. It can be seen from the XRD spectra that the catalysts have no obvious peak, indicating that the surface morphology of the supported catalyst is mainly amorphous. A broad peak appeared at 2θ = 15.0°–27.8°, which is mainly attributed to the amorphous silica. Furthermore, the fine diffraction peak is attributed to the special structure of the organic/inorganic hybrid zirconium phosphate. Theoretically, each of the composite zirconium salt particles is composed of a plurality of layered composite zirconium salt crystallites, and the arrangement of the crystal planes of the respective zirconium salt crystal grains is randomly arranged. When the homogeneous chiral Mn^III^ (salen) complex catalyst is fixed, it is distributed on the surface of the layer or between the layers. Therefore, the catalyst results are very likely to present a form of ‘short-range order, long-range disorder’.

### 4.4. Microscopic Analysis

**Figure 4:**
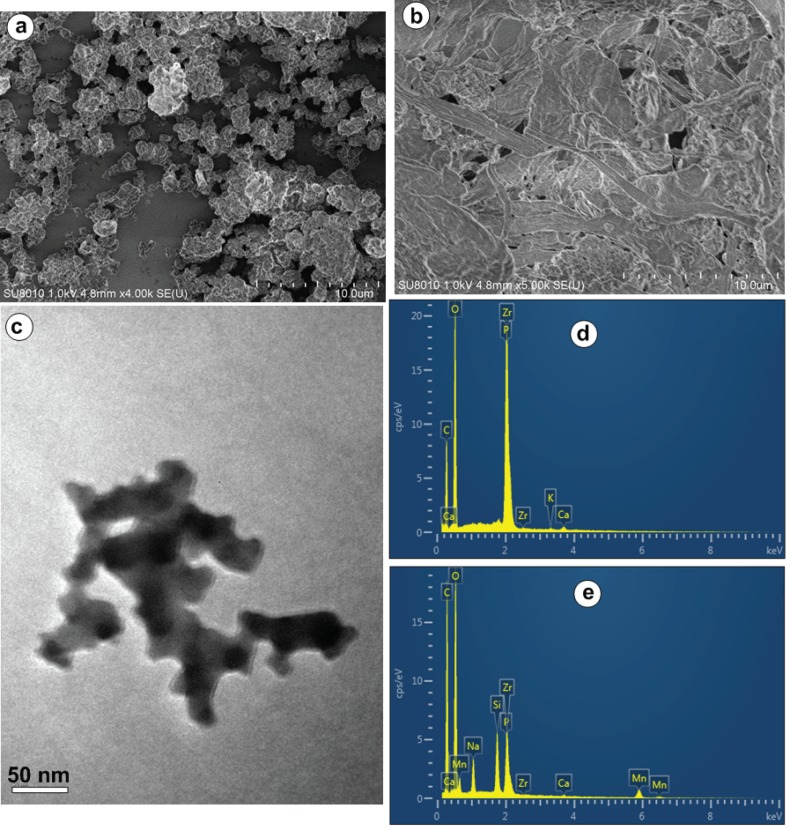
SEM photograph of (**a**) ZPS-PVPA, (**b**) catalyst 3, TEM photograph of (**c**) catalyst 3, and the measured EDS image of (**d**) ZPS-PVPA, (**e**) catalyst 3.

SEM images provide direct information on the microstructure and morphology of ZPS-PVPA and catalyst 3, as shown in Figure 4. Figure 4a indicates that the amorphous ZPS-PVPA was loose, and various cavities, holes, and pores were present in every particle. Figure 4b indicates that the catalyst structure contains channels of different shapes and sizes. The TEM image of the heterogeneous catalyst 3 (Figure 4c) indicates that the configuration of the catalyst exhibits a completely amorphous and disordered structure. Compared with Figure 4d, a new Si peak and Mn peak appear in Figure 4e, which fully demonstrates that ZPS-PVPA is functionalized by amine groups, and that the chiral Mn^III^ (salen) is successfully immobilized by axial means.

**Figure 5:**
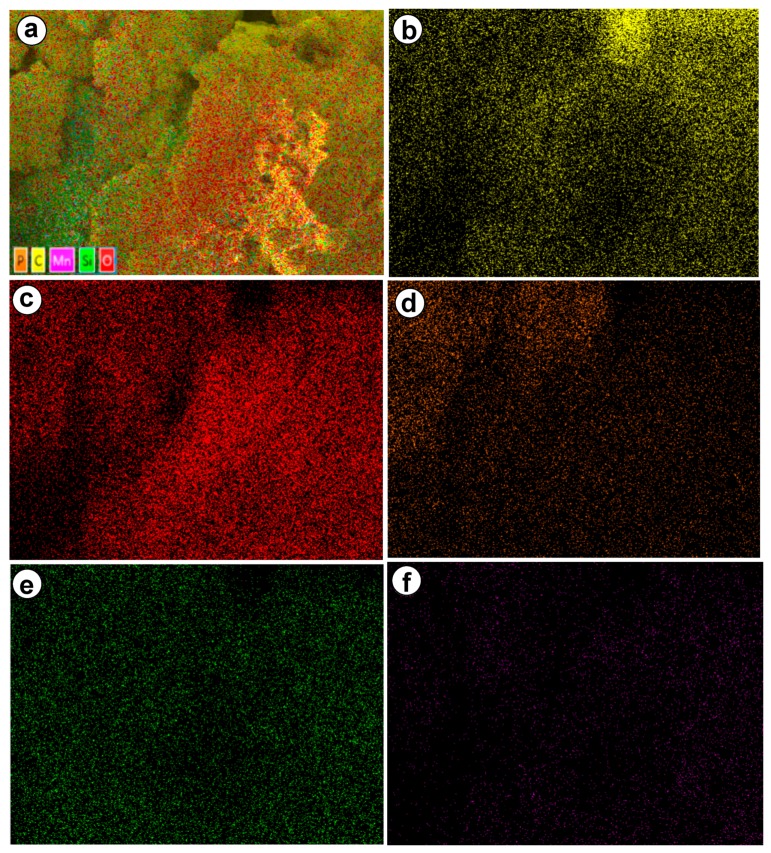
EDX elementary mapping of (**a**) layered image of catalyst 2, (**b**) C, (**c**) O, (**d**) P, (**e**) Si, and (**f**) Mn in catalyst 3.

To confirm the existence of Mn^III^ (salen) complex on/in the ZPS-PVPA, we carried out element mapping of catalyst 3 (Figure 5a–e). Considerable quantities of Mn and Si were detected along with Zr, P, and O, implying the existence Mn^III^ (salen) complex on/in the ZPS-PVPA.

### 4.5. X-ray Photoelectron Spectroscopy

**Figure 6:**
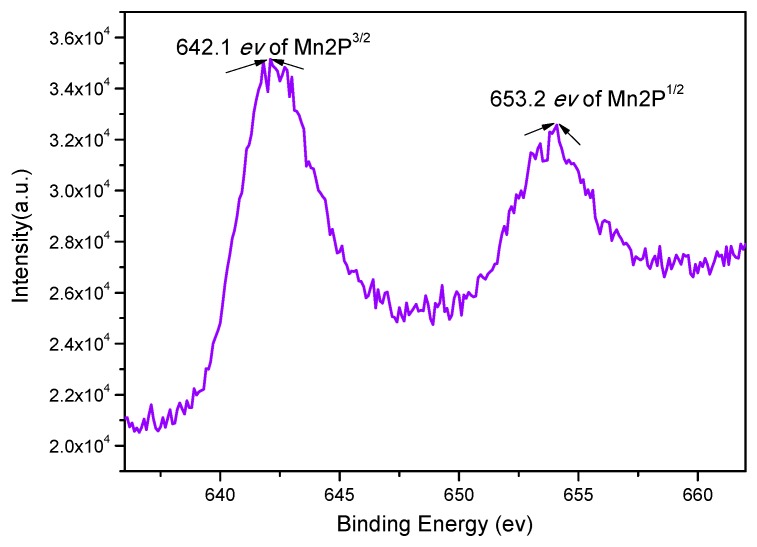
X-ray photoelectron spectroscopy (XPS) spectra of catalyst 3.

X-ray photoelectron spectroscopy (XPS) is extensively used to obtain detailed information about the state of metal species on surfaces, and to investigate the electronic properties of the species formed on the surface [20]. Figure 6 shows the Mn 2P XPS spectrum of catalyst 3. There are major peaks with binding energies of 642.1 and 653.2 *eV*, which correspond to Mn 2 P^3/2^ and 2P^1/2^. A similar shift has also been reported earlier, where chiral salen Mn^III^ catalysts and Schiff bases were immobilized on an amine modified ZPS-PVPA through N–Mn bonding [17,21].

### 4.6. Enantioselective Epoxidation of Styrene

The catalytic tests for the heterogeneous chiral Mn^III^ (salen) complex were compared with the corresponding homogeneous catalyst under identical reaction conditions. In this work, styrene was chosen as the model substrate to evaluate the activity of heterogeneous catalysts 1–3. The results are summarized in Table 1. The results show that the heterogeneous catalysts efficiently promoted the enantioselective epoxidation of styrene (entries 2, 3, and 4), as the *ee* obtained is higher than that of the corresponding homogeneous catalyst (*ee*%, 53.3–63.9 vs. 46.2, Table 1). The increase in chiral recognition is mainly attributed to the microenvironment effects of the organic polymer–inorganic zirconium hydrogen phosphate immobilized Mn^III^ (salen) [15,16,17,18,19]. The microenvironment effects originated from the layered structure, micoropores, and channels, and the hydrophilic property of the polystyrenylphosphonate parts and hydrophobic property of the zirconium parts of the hybrid zirconium phosphonate. These features are different from either pure polystyrene or pure zirconium hydrogen phosphate. In absence of NMO, only 3.9% *ee* values and 69.6% conversions were obtained (entry 5, Table 1), suggesting that the presence of NMO facilitates higher catalytic activity and enantioselectivity. The role of NMO as an axial base was reported earlier by our group [15,16,17], as well as by other groups [22]. ZAPS-PVPA alone showed negligible catalytic activity toward the epoxidation of styrene (*conv*%: 12.3, entries 6, Table 1). Furthermore, catalyst 3 was found to be more efficient than catalysts 1 and 2 in the epoxidation of styrene under identical reaction conditions. It was deduced that the increase in catalytic efficiency is mainly attributed to the carrier effect. Compared to the carriers ZSPP and ZPS-IPPA, ZPS-PVPA has a larger specific surface area, pore volume, and pore size [15,16,17,23,24]. In the process of precipitation of poly(styrene-styrene)phosphonic acid and inorganic zirconium phosphate, poly(styrene-styrene) is bulky and has a high degree of polymerization. Once more of the –PO_3_H_2_ groups are brought together, the polystyrene organic segments in the copolymer can aggregate and join to form channels, pores, and cavities of different pore sizes and shapes. It is these micro-environments formed by channels, pores, and cavities of different pore sizes and shapes and the special layered structure of inorganic zirconium hydrogen phosphate which ultimately lead to differences in the catalytic efficiency of the supported catalysts.

In order to determine the optimal amount of catalyst 3, the reaction was carried out in the presence of different amounts (0.5–5 mol%, based on Mn element) at 0 °C. The results showed that the conversions were increased from 35.6 % to 95.0%, with an increase in *ee* valules from 49.6% to 64.7% when the amount of catalyst was increased from 0.5 to 3 mol%. Moreover, further increasing the amount of catalyst did not show any positive effect on the conversion and *ee* valules of the styrene epoxide (Table 2, entry 5–6). Blank experiments showed that catalyst 3 alone is inactive towards epoxidation of styrene (Table 2, entry 7). Similar results have been reported in the epoxidation of *α*-methylstyrene catalyzed by GO-salenMn [13].

Table 3 summarizes the results of a comparative study of the enatioselective epoxidation of styrene with catalyst 3 in different solvents. As expected, the catalytic activity and enantioselective property were related to the solvents. It was found that dichloromethane was the optimal solvent (*conv*%: 95.0, *ee*%: 64.7) for the reaction, while only a 41.9 conversion with a 13.9% *ee* values and a 40.1% conversion with a 15.5% *ee* values were obtained when the reaction was performed in *n*-hexane and alcohol, respectively. The difference among these solvents is likely that catalyst 3 has a better swelling in the solvent dichloromethane than in other mediums such as hexane. The *ee* values obtained in ethyl acetate, acetonitrile, and acetone were lower than in dichloromethane, which indicates that the oxygen and nitrogen atoms with a lone electron pair can induce coordination with the metal center of the chiral Mn^III^ (Salen) complex, suppressing formation of the active oxygen transfer species (Mn–*V*–oxo) in the epoxidation of styrene [25,26]. In particular, an *ee* of only 31.5% was obtained in acetonitrile.

### 4.7. Effect of Reaction Temperature

Table 4 summarizes the catalytic activity of catalyst 3 at different reaction temperatures. It was found that the conversions decreased from 95.0% to 70.6% when the reaction temperature decreased from 0 to −78 °C, while an increase of enantioselectivity (from 64.7% to 73.9%) was observed, which was similar to the results reported previously [27]. The reason is likely due to both an increase in enantiofacial selectivity in the initial C−O bond forming step, and suppression of the trans-pathway in the second step at low temperature [28]. It was encouraging that an enantioselectivity as high as 73.9% could be obtained when the reaction was performed at −78 °C. However, from a practical point of view, the results are meaningless.

### 4.8. The Recycling of the Supported Chiral Mn^III^ (Salen) Catalyst

To assess the recyclability of the heterogeneous catalyst, catalyst 3 was completely washed with ethanol and dichloromethane and used in repeated epoxidation reactions with styrene by adding fresh reactants. In each run, no manganese was detected in the filtrates by AAS, which indicates that the Mn^III^ (salen) complex was strongly bonded onto the amine modified ZPS-PVPA supports. The catalytic results are presented in Table 5. It is obvious that there was no significant decrease in catalytic activity after catalyst 3 was recycled four times. (*cons*%: from 95.0 to 92.6; *ee*%: from 64.7to 60.1). Further recycles of the heterogeneous catalyst resulted in poor conversions, although the enantioselectivity obtained was still higher than that of the corresponding homogeneous catalyst even after eight recycles. We think that the solid catalyst has a micro-environment composed of pores, voids, and secondary channels of different sizes, which is the main reason why catalyst 3 exhibits excellent cycle performance. However, the catalytic performance of the cycle was gradually decreased in the following runs. It is possible that the space structure of the channels, pores, and cavities of the catalyst itself were also damaged to some degree, or that the secondary channels and micropores were blocked, so that the active center of the catalyst was slightly affected, which in turn affected the final catalytic performance of the catalyst.

However, after the end of the eighth reaction, the solid catalyst was allowed to stand in 2 mol/L of dilute hydrochloric acid for overnight, which resulted in the unexpected discovery that the catalytic activity of the catalyst was recovered again at the 9th and 10th cycles of the catalyst. Due to the nano-layered, self-supporting function of the inorganic zirconium phosphate portion of the catalyst structure, the catalyst may have a certain ‘memory function’, and can be roughly restored to the original morphology [29].

## 5. Conclusions

The heterogeneous catalysts were prepared by heterogenizing asymmetric chiral Mn^III^ (salen) complex onto 3-aminopropyltrimethoxysilane-functionalized modified organic polymer–inorganic zirconium hydrogen phosphate (ZSPP, ZPS-IPPA, and ZPS-PVPA) by an axially bonding method. The prepared catalysts effectively catalyzed epoxidation of styrene (*ee*%, 53.3–63.9) with *m*-CPBA/NMO as oxidant. The results demonstrated that the *ee* values obtained were higher than that of a corresponding homogeneous catalyst (*ee*%, 53.3–63.9 *vs*. 46.2). Moreover, catalyst 3 was found to be more efficient than catalysts 1 and 2 under identical reaction conditions. In addition, it is obvious that there was no significant change in catalytic activity after the catalyst 3 was recycled four times (*cons*%: from 95.0 to 92.6; *ee*%: from 64.7 to 60.1). Further recycles of the heterogeneous catalyst resulted in poor conversions, although the enantioselectivity obtained was still higher than that of the corresponding homogeneous catalyst even after eight recycles. After the end of the eighth reaction, the solid catalyst was allowed to stand in 2 mol/L of dilute hydrochloric acid for overnight, after which an unexpected discovery was made that the catalytic activity of the catalyst was recovered again at the 9th and 10th cycles of the catalyst. However, the reasons remain to be further studied.

## Figures and Tables

**Table 1:** Conversions and *ee* values for enantioselective for epoxidation of styrene catalyzed by chiral Mn^III^ salen and catalysts 1–3 ^a^.

Entry	Catalyst	Oxidant	Conv (%(h)) ^b^	Sele (%) ^c^	Ee (%) ^c^
1	Chiral Mn^III^ (salen)Cl	*m*-CPBA/NMO	99.9(1)	98.7	46.2 ^d^
2	Catalyst 1	*m*-CPBA/NMO	92.6(3)	91.7	53.3 ^d^
3	Catalyst 2	*m*-CPBA/NMO	93.8(3)	94.3	57.9 ^d^
4	Catalyst 3	*m*-CPBA/NMO	95.1(3)	99.1	63.9 ^d^
5	Catalyst 3	*m*-CPBA	69.6(3)	81.7	3.90 ^d^
6	ZAPS-PVPA	*m*-CPBA	12.3(3)	-	-

^a^ Reactions were carried out at 0 °C in CH_2_Cl_2_ (3 mL) with alkene (0.5 mmol), *m*-CPBA (1 mmol), NMO (337.5 mg, 2.5 mmol, if necessary), nonane (internal standard, 56 mL, 0.5 mmol), and the homogeneous (5 mol%) or heterogeneous Mn^III^ salen catalysts (0.015 mmol, 3.0 mol%, based on the Mn element). ^b^ Conversions were determined by gas chromatography (GC), by integration of product peaks against an internal quantitative standard (nonane), correcting for response factors. ^c^ Determined by GC with a chiral capillary column (HP19091G-B233, 30 m × 0.25 mm × 0.25 μm). ^d^ Epoxide configuration R.

**Table 2:** The results of screening the quantity of catalyst 3 in the epoxidation of styrene ^a^.

Entry	Catalyst mol%	Conv (%(h)) ^b^	Sele (%) ^c^	Ee (%) ^c^
1	0.5	35.6(3)	78.3	49.6 ^d^
2	1	50.9(3)	80.1	56.6 ^d^
3	2	82.6(3)	88.7	60.3 ^d^
4	3	95.0(3)	96.9	64.7 ^d^
5	4	95.1(3)	97.6	63.1 ^d^
6	5	95.1(3)	99.1	63.9 ^d^
7	No catalyst	7.9(3)	-	-

^a^ same as in Table 1. ^b^ same as in Table 1. ^c^ same as in Table 1. ^d^ same as in Table 1.

**Table 3:** The results of asymmetric epoxidation of styrene in different solvents over catalyst 3 ^a^.

Entry	Solvent	Conv (%(h)) ^b^	Sele (%) ^c^	Ee (%) ^c^
1	Dichloromethane	95.0(3)	96.9	64.7 ^d^
2	n-Hexane	41.9(3)	27.6	13.9 ^d^
3	ethanol	40.1	18.6	15.5 ^d^
4	Ethyl acetate	89.9	83.6	41.0 ^d^
5	Acetone	83.2	80.6	38.2 ^d^
6	Acetonitrile	90.6	91.8	31.5 ^d^

^a^ same as in Table 1. ^b^ same as in Table 1. ^c^ same as in Table 1. ^d^ same as in Table 1.

**Table 4:** The results of asymmetric epoxidation of styrene in different temperatures over catalyst 3 ^a^.

Entry	Temperature (°C)	Conv (%(h)) ^b^	Sele (%) ^c^	Ee (%) ^c^
1	0	95.0(3)	96.9	64.7 ^d^
2	−20	86.8(2)	85.2	65.2 ^d^
3	−40	79.2(2)	86.3	67.8 ^d^
5	−78	70.6(1)	88.1	73.9 ^d^

^a^ same as in Table 1. ^b^ same as in Table 1. ^c^ same as in Table 1. ^d^ same as in Table 1.

**Table 5:** The recycling of catalyst 3 in the asymmetric epoxidation of styrene with *m*-CPBA/NMO as oxidants ^a^.

Run	Conv (%(h)) ^b^	Sele (%) ^c^	Ee (%) ^c^
1	95.0(3)	96.9	64.7 ^d^
2	95.0(3)	93.8	62.9 ^d^
3	94.1(3)	89.6	62.1 ^d^
4	92.6(3)	81.6	60.1 ^d^
5	91.2(3)	71.8	58.7 ^d^
6	85.8(3)	64.6	55.1 ^d^
7	80.8(3)	50.9	51.3 ^d^
8	66.8(3)	43.2	48.1 ^d^
9	93.2(3)	86.0	54.0 ^d^
10	92.8(3)	85.1	52.3 ^d^

^a^ same as in Table 1. ^b^ same as in Table 1. ^c^ same as in Table 1. ^d^ same as in Table 1.
